# Supplementary material for: Pyruvate Dehydrogenase Kinase 1 inhibition mediated oxidative phosphorylation enhancement in cartilage promotes osteoarthritis progression
Source: BMC Musculoskelet Disord. 2023 Jul 20;24:597. doi: 10.1186/s12891-023-06585-6 (PMC10357736; doi:10.1186/s12891-023-06585-6)
Supplement: Supplementary file 1 — Additional file 1. [file 12891_2023_6585_MOESM1_ESM.zip › MF.Gsea.1653627316242/GOMF_RIBOSOMAL_SMALL_SUBUNIT_BINDING.html]

Details for gene set GOMF\_RIBOSOMAL\_SMALL\_SUBUNIT\_BINDING[GSEA]

|  || Dataset | 55457\_collapsed\_to\_symbols.cls.cls #OA\_versus\_normal.cls.cls #OA\_versus\_normal\_repos |
| Phenotype | cls.cls#OA\_versus\_normal\_repos |
| Upregulated in class | normal |
| GeneSet | GOMF\_RIBOSOMAL\_SMALL\_SUBUNIT\_BINDING |
| Enrichment Score (ES) | -0.55550396 |
| Normalized Enrichment Score (NES) | -1.5623369 |
| Nominal p-value | 0.030909091 |
| FDR q-value | 0.15168178 |
| FWER p-Value | 0.999 |
Table: GSEA Results Summary

  

Fig 1: Enrichment plot: GOMF\_RIBOSOMAL\_SMALL\_SUBUNIT\_BINDING      
 Profile of the Running ES Score & Positions of GeneSet Members on the Rank Ordered List

  

| SYMBOL | TITLE | RANK IN GENE LIST | RANK METRIC SCORE | RUNNING ES | CORE ENRICHMENT || 1 | DHX29 | DExH-box helicase 29 [Source:HGNC Symbol;Acc:HGNC:15815] | 1012 | 0.515 | 0.0125 | No |
| 2 | MTIF2 | mitochondrial translational initiation factor 2 [Source:HGNC Symbol;Acc:HGNC:7441] | 1043 | 0.508 | 0.1001 | No |
| 3 | EIF4H | eukaryotic translation initiation factor 4H [Source:HGNC Symbol;Acc:HGNC:12741] | 5118 | 0.062 | -0.2050 | No |
| 4 | UNG | uracil DNA glycosylase [Source:HGNC Symbol;Acc:HGNC:12572] | 6078 | -0.011 | -0.2776 | No |
| 5 | ERAL1 | Era like 12S mitochondrial rRNA chaperone 1 [Source:HGNC Symbol;Acc:HGNC:3424] | 6158 | -0.017 | -0.2807 | No |
| 6 | NME1 | NME/NM23 nucleoside diphosphate kinase 1 [Source:HGNC Symbol;Acc:HGNC:7849] | 6328 | -0.031 | -0.2884 | No |
| 7 | EIF1B | eukaryotic translation initiation factor 1B [Source:HGNC Symbol;Acc:HGNC:30792] | 7846 | -0.144 | -0.3807 | No |
| 8 | EIF4B | eukaryotic translation initiation factor 4B [Source:HGNC Symbol;Acc:HGNC:3285] | 8051 | -0.159 | -0.3684 | No |
| 9 | PTCD3 | pentatricopeptide repeat domain 3 [Source:HGNC Symbol;Acc:HGNC:24717] | 8287 | -0.178 | -0.3552 | No |
| 10 | ABCE1 | ATP binding cassette subfamily E member 1 [Source:HGNC Symbol;Acc:HGNC:69] | 9675 | -0.289 | -0.4117 | No |
| 11 | LARP1 | "La ribonucleoprotein 1, translational regulator [Source:HGNC Symbol;Acc:HGNC:29531]" | 10964 | -0.425 | -0.4364 | Yes |
| 12 | PIM1 | "Pim-1 proto-oncogene, serine/threonine kinase [Source:HGNC Symbol;Acc:HGNC:8986]" | 12500 | -0.748 | -0.4231 | Yes |
| 13 | NPM1 | nucleophosmin 1 [Source:HGNC Symbol;Acc:HGNC:7910] | 12530 | -0.762 | -0.2906 | Yes |
| 14 | EIF1 | eukaryotic translation initiation factor 1 [Source:HGNC Symbol;Acc:HGNC:3249] | 12690 | -0.855 | -0.1517 | Yes |
| 15 | DDX3X | DEAD-box helicase 3 X-linked [Source:HGNC Symbol;Acc:HGNC:2745] | 12788 | -0.949 | 0.0087 | Yes |
Table: GSEA details [plain text format]

  

Fig 2: GOMF\_RIBOSOMAL\_SMALL\_SUBUNIT\_BINDING      
 Blue-Pink O' Gram in the Space of the Analyzed GeneSet

  

Fig 3: GOMF\_RIBOSOMAL\_SMALL\_SUBUNIT\_BINDING: Random ES distribution      
 Gene set null distribution of ES for **GOMF\_RIBOSOMAL\_SMALL\_SUBUNIT\_BINDING**
